# *In vitro* and *ex vivo* testing of alternative disinfectants to currently used more harmful substances in footbaths against *Dichelobacter nodosus*

**DOI:** 10.1371/journal.pone.0229066

**Published:** 2020-02-13

**Authors:** Tobias Hidber, Urs Pauli, Adrian Steiner, Peter Kuhnert

**Affiliations:** 1 Institute of Veterinary Bacteriology, Vetsuisse Faculty, University of Bern, Bern, Switzerland; 2 Clinic for Ruminants, Vetsuisse Faculty, University of Bern, Bern, Switzerland; 3 Institute of Virology and Immunology, Mittelhäusern, Köniz, Switzerland; Veterinary Pathology, SWITZERLAND

## Abstract

A footbath-based control program for ovine footrot, a contagious disease caused by *Dichelobacter nodosus*, will be implemented in Switzerland. The currently used footbath disinfectants formaldehyde, zinc sulfate and copper sulfate are carcinogenic or environmental pollutants. Hence, the aim of this study was to identify alternative disinfectants, which are highly effective, non-carcinogenic, environmentally acceptable, inexpensive, available as concentrate and suitable for licensing. The antimicrobial effect of a series of potential chemicals such as lactic acid, propionic acid, hydrogen peroxide, sodium hypochlorite, octenidine dihydrochloride, chlorocresol, Ampholyt 20 and the registered biocide DESINTEC® Hoof Care Special D (Desintec) were investigated by culture based *in vitro* testing. The microcidal effect of various Desintec concentrations were then compared against routinely used 4% formaldehyde and 10% zinc sulfate in *ex vivo* assays on sheep feet from slaughter. For this purpose a newly established PMA (propidium monoazid) real-time PCR using the improved dye PMAxx^™^ was applied that allows discrimination of viable and dead *D*. *nodosus*. In the *ex vivo* experiments, 4% formaldehyde was significantly more effective than 10% zinc sulfate and was chosen as positive control for assessing the new disinfectant. The disinfectant effect of Desintec in a minimal concentration of 6% was equally effective as 4% formaldehyde, meaning that it offers a comparable antimicrobial effect against virulent *D*. *nodosus*. In conclusion, Desintec is a promising disinfectant for replacing formaldehyde, copper sulfate and zinc sulfate in footbaths against footrot.

## Introduction

*Dichelobacter nodosus* is a gram-negative fastidious anaerobic bacterium and the causative agent of ovine footrot [1]. The disease has a global presence and is endemic in many countries [2]. In Switzerland, the true prevalence of virulent *D*. *nodosus* in sheep on animal level is estimated at 16.9% and on farm level at 16.2% [3]. Clinical symptoms range from mild interdigital dermatitis in benign footrot to severe underrunning and separation of the hoof horn from the underlying tissue in virulent footrot. Clinical symptoms start as early as 2 weeks after first contact and the disease leads to pain, lameness, decreased meat and wool production as well as animal welfare issues [4–6]. The economic and intangible costs of the disease are considerable. In Switzerland, costs for management and growth reduction without control measures were estimated at CHF 172.3 million for 2014–2030 [7]. To address these problems, various countries started to develop and implement footrot control or elimination programs [3].

In Switzerland, a mandatory footrot control program started first in the canton of Grisons in 1994 whereas in other cantons sheep owners could voluntarily join the control program offered by the Swiss Consulting and Health Service for Small Ruminants. The successful control in Grisons, progress in laboratory diagnostics allowing PCR-detection and discrimination of virulent and benign *D*. *nodosus* [8], and the ongoing unsatisfactory situation in other cantons led to the political decision for a nationwide footrot control program. A cost-benefit analysis confirmed positive epidemiological and economic effects of this approach [7]. The Federal Food Safety and Veterinary Office is currently preparing the nationwide footrot control program, which is planned to start in 2022. The goal is to reduce flock prevalence of virulent *D*. *nodosus* to less than one percent within five years.

The control program consists of three phases: i) swab sampling for detection of virulent (*aprV2*-positive) *D*. *nodosus* by PCR, ii) treatment of *aprV2*-positive herds by claw-trimming and weekly footbaths in disinfectant solution, iii) surveillance of treated herds.

Most frequently used disinfectants in footbaths in Switzerland are 4% formaldehyde, 10–20% zinc sulfate and 5–10% copper sulfate [9]. In a recent proof-of-concept study, weekly footbaths in 10% zinc sulfate was shown to eliminate *aprV2*-positive *D*. *nodosus* from feet of sheep within 6–19 weeks [10]. Despite their effectiveness, these substances have undesirable characteristics for use in a nationwide control program. Formaldehyde smells pungent and irritates airways. It is a known cause of allergic contact dermatitis and a carcinogen in both humans and animals [11]. Repeated use of formaldehyde footbaths in sheep caused keratinization of the interdigital skin, which can lead to secondary infection and lameness [12]. Zinc and copper are both essential trace elements, acting as catalytic or structural components of larger molecules and are therefore indispensable for live. However, similar to more toxic heavy metals they are a major contaminant of soil and groundwater, accumulating in water, sediment, aquatic plants and fishes, posing a potential health threat to aquatic, human and animal life [13].

Hence, the aim of this study was to identify an alternative disinfectant solution, which is highly effective against *D*. *nodosus*, non-carcinogenic, environmentally acceptable, inexpensive, available as concentrate and suitable for licensing as biocide for treating footrot in Switzerland. The effects of different disinfectant solutions on virulent *D*. *nodosus* were investigated by culture based *in vitro* testing and *ex vivo* evaluation applying a newly established PMA (propidium monoazid) real-time PCR (PMA-qPCR) using the improved dye PMAxx^™^ allowing discrimination of viable and dead *D*.*nodosus*.

## Materials and methods

### *In vitro* disinfectant testing

Disinfectants were selected from literature considering their toxicity, degradability and availability as concentrate. Three disinfectants already in use for treatment of footrot in Switzerland and 19 substances or products selected by expert opinion based on the results of a literature search were evaluated *in vitro* for their antimicrobial activity against virulent *D*. *nodosus* (Table 1). Disinfectants, which resulted in a ≥ 5 log reduction of the number of colony-forming units (CFU), were further tested with organic soiling. Formaldehyde, copper sulfate, zinc sulfate, DESINTEC® Hoof Care Special D (Desintec) and its main compounds acetic acid, glycolic acid and glutaraldehyde were tested three times, all other substances once, with and without simulating soiling. The composition of Desintec is given in Table 1.

**Table 1:** Disinfectants tested.

Trade name / Active ingredient	Manufacturer	CAS-No^a^
Formaldehyde	Sigma-Aldrich	50-00-0
Copper sulfate	Sigma-Aldrich	7758-99-8
Zinc sulfate	Sigma-Aldrich	7446-19-7
DESINTEC® Hoof Care Special D	FINK TEC (PediSept G20)	
Acetic acid (10.0%)		64-19-7
Glycolic acid (8.8%)		79-14-1
Glutaraldehyde (6.0%)		111-30-8
Fatty alcohol ethoxylate (< 2.5%)		68439-50-9
Aluminium sulfate (<2.5%)		10043-01-3
Water		7732-18-5
Acetic acid	Sigma-Aldrich	64-19-7
Glycolic acid	Sigma-Aldrich	79-14-1
Glutaraldehyde	Sigma-Aldrich	111-30-8
L-Lactic acid	Sigma-Aldrich	79-33-4
Sodium benzoate	Sigma-Aldrich	532-32-1
Propionic acid	Sigma-Aldrich	79-09-4
Tartaric acid	Sigma-Aldrich	87-69-4
Calcium magnesium oxide	Kalkwerk Hufgard	37247-91-9
Calcium magnesium tetrahydroxide	Kalkwerk Hufgard	39445-23-3
Calcium hydroxide	Sigma-Aldrich	1305-62-0
Calcium oxide	Sigma-Aldrich	1305-78-8
Desical ® plus	Kalkwerk Hufgard	
Calcium magnesium oxide		37247-91-9
Calcium magnesium tetrahydroxide		39445-23-3
Hydrogen peroxide	Sigma-Aldrich	7722-84-1
Sodium hypochlorite	Sigma-Aldrich	7681-52-9
Toucan Eco ®		
(electrochemically activated water)	Green Innovations	-
Octenidine dihydrochloride	Ark Pharm	70775-75-6
Chlorocresol	Sigma-Aldrich	59-50-7
Tego ® 2000 VT25 **(**Ampholyt 20)	Diversey, Sealed Air Food Care	139734-65-9

^a^ CAS = Chemical Abstracts Service

Virulent (*aprV2*-positive) *D*. *nodosus* ATCC 25549^T^ was cultured on Brucella Blood Agar with Hemin and Vitamin K1 (Becton Dickinson) at 37°C in an anaerobic chamber (80% N_2_, 10% CO_2_ and 10% H_2_; Scholzen Microbiology Systems AG). After 4–5 days, cultures were transferred with a cotton swab into Difco^™^ LB Broth (Becton Dickinson) and suspended until McFarland 4 was reached in Densichek (bioMérieux). To simulate high-level soiling, a solution of 10% Bovine Serum Albumin (Sigma-Aldrich) and 10% yeast extract (Becton Dickinson) was filtered through a 0.2 μm Acrodisc syringe filter (Pall Corporation) and complemented with 10% defibrinated sheep blood (Thermo Fisher Scientific) resulting in a 10x soiling-solution.

To determine the CFU/ml in the test mixture at the beginning of contact time (*N*_*0*_*)*, the number of CFU/ml in the test mixture after disinfectant treatment at the end of contact time (*N*_*D*_), and in the positive control, 1 ml of LB or 1 ml soiling-solution were prepared in a 15 ml Falcon tube (Sarstedt). Then 8 ml of LB, 8 ml 1.25x disinfectant solution or 8 ml 5% formaldehyde (Sigma-Aldrich) were added, respectively. Finally, 1 ml of *D*. *nodosus* suspension was added before mixing the tubes. After 5 min contact time at room temperature, the tubes were centrifuged for 5 min at 4255xg, the supernatant discarded and the pellets resuspended in 10 ml LB. This was repeated twice, the last time with resuspension in 1 ml LB. Subsequently, tenfold dilution series down to 10^−7^ were prepared with 100 μl test suspension and 900 μl LB out of which 500 μl were plated on Brucella Blood Agar. The plates were incubated for 4–5 days as described before.

After counting, plates with CFU in the range of 14 to 330 were included for calculation of the weighted mean count of *N*_*0*_ and *N*_*D*_. If possible, two dilutions were evaluated, otherwise only one.

*N*_*0*_ and *N*_*D*_ were calculated as follows:

$$N=\frac{c}{(n_{1}+0.1n_{2})vd}$$

where

*c* is the sum of CFU taken into account;

*n*_*1*_ is the number of plates taken into account in the lower dilution;

*n*_*2*_ is the number of plates taken into account in the higher dilution;

*v* is the volume plated in ml;

*d* is the dilution factor corresponding to the lower dilution.

Example of two dilutions:

$$N=\frac{244+33}{(1+0.1\cdot 1)\cdot 0.5\cdot 10^{-5}}=\frac{277}{1.1\cdot 0.5\cdot 10^{-5}}=5.0\cdot 10^{7}\;(in\;CFU/ml)$$

The reduction (R) was expressed as decimal logarithm: log_10_R = log_10_ *N*_*0*_ –log_10_ *N*_*D*_. Whenever *N*_*D*_ was zero, the value "1" was applied. Disinfectants demonstrating a ≥ 5 log_10_ reduction at 5% disinfectant concentration with and without soiling were considered useful [14, 15].

### *Ex vivo* experiments

#### PMAxx^™^ treatment conditions and linear range of PMA-qPCR

A 1 ml McFarland 4 suspension of virulent *D*. *nodosus* was prepared as described before. Half of the suspension was heated at 99°C for 10 min. With both, the living and heat-treated *D*. *nodosus* suspension, tenfold serial dilutions were prepared down to 10^−5^.

For determining CFU in the suspension, 10 μl of each untreated 10^−3^, 10^−4^ and 10^−5^ dilution were mixed with 500 μl 0.85% NaCl, plated on Brucella Blood Agar and incubated for 4–5 days. Killing of heat-treated *D*. *nodosus* was confirmed by plating 10 μl of the heated-treated suspension in the same way.

For PMA-qPCR enumeration of viable and dead *D*. *nodosus* in the dilution series, 10 μl of dilution were mixed with 40 μl of 0.85% NaCl in transparent 1.5 ml tubes (Sarstedt). In a darkened room 100 μM (0.25 μl) of PMAxx^™^ (Biotium) was added and the tube was vortexed and incubated for 3 min at room temperature in a metal box impervious to light. After incubation, tubes were placed on ice and exposed to LED light (Optonica LED floodlight Item No. FL5836, white light, 100 W, 6000 K, 8500 lm) at a distance of 20 cm for 5 min. Following light treatment, the tubes were centrifuged at 15'000xg for 5 min in a microcentrifuge. To remove the supernatant, the tubes were held in a horizontal position and twisted tissues were used to absorb the liquid. The pellets were resuspended in 500 μl SV-lysis buffer (4 M guanidine thiocyanate, 0.01M Tris–HCl, 1% β-mercaptoethanol).

DNA extraction was done following established protocols [5] including the VetMax^™^ Xeno^™^ IPC (Thermo Fisher Scientific) on an automated purification system (KingFisher^™^ Duo-Prime, Thermo Fisher Scientific). Extracted DNA was used directly or stored at -20°C until further qPCR analysis was undertaken.

Genomic DNA of *D*. *nodosus* ATCC 25549^T^ was used as an external standard in the qPCR with seven points corresponding from 10^7^ to 10^1^ genome equivalents per well. Dilution stages of live and dead *D*. *nodosus* were analyzed in duplicate, the external standard in triplicate. Assay conditions consisted of a 25 μl reaction mixture containing 1 x TaqMan^™^ Fast Advanced Master Mix (Thermo Fisher Scientific), 300 nM primers DnAprTM-L and DnAprTM-R, 100 nM Probe DnAprTm-v, 250 nM Probe DnAprTM-b, pyrogen-free water, 1 μl Xeno^™^ LIZ Primer Probe Mix (Thermo Fisher Scientific) and 2.5 μl of DNA template. Amplification was done in a 7500 Real-Time PCR-System instrument (Applied Biosystems), using cycles of 2 min at 50°C and 10 min at 95°C followed by 40 cycles with 15 s at 95°C and 1 min at 60°C. Results were analyzed using the 7500 Software (v 2.3.) with the threshold set at 0.015 [8]. Mean cycle threshold (Ct) values of duplicates and triplicates were calculated. The Ct values of heat-treated and live *D*. *nodosus* dilution series were plotted versus the log quantity of standard DNA. Determination of linear range was done three times and average values calculated.

The protocol for detection of live *D*. *nodosus* by PMA-qPCR is publicly available at http://dx.doi.org/10.17504/protocols.io.bbh9ij96

#### *Ex vivo* disinfectant testing

**Fig 1:**
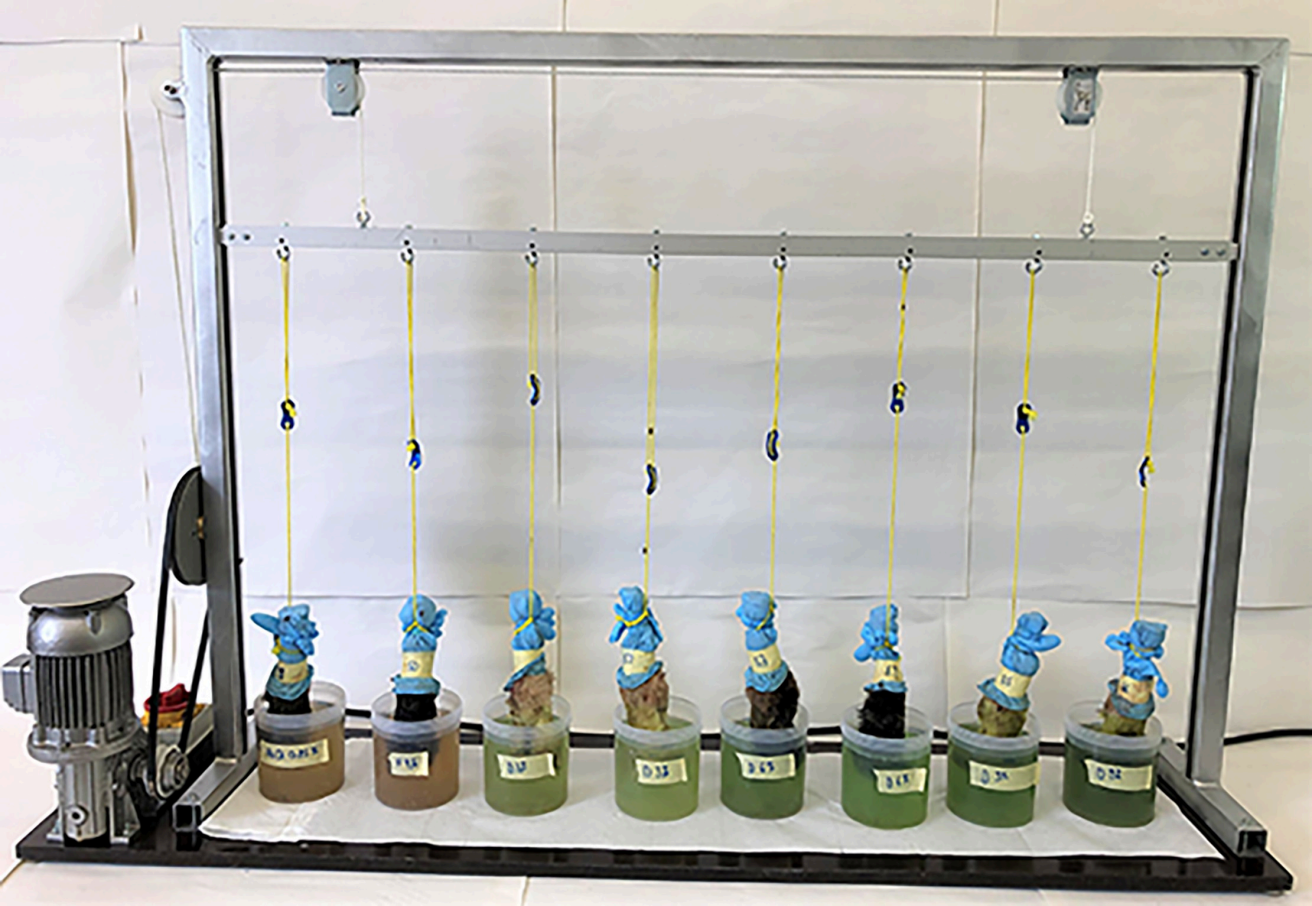
Foot-dipping machine. The foot-dipping machine simulates the movement of alive sheep feet in disinfectant footbath solution.

Clinically affected feet from sheep with footrot score ≥ 2 were collected at the abattoir in Thun and transported at room temperature to the laboratory within 30 min. Gross manure was cautiously removed from claws and the interdigital space, the open articulation of the carpal or tarsal joint was covered with gloves and an identification number was assigned to each foot. A cotton swab was soaked in 0.85% NaCl and a first sample (prevalue) was taken from the interdigital space. The swab was once rotated 360 degrees and subsequently soaked in 500 μl of 0.85% NaCl in a 1.5 ml tube. The feet were then attached to the cords of an in-house foot-dipping machine, which allowed foot-dipping of eight feet in parallel and had a frequency of five down movements per minute and a total contact time with the disinfectant of 50 s per minute (Fig 1).

The machine was turned on, and feet were dipped and moved in plastic beakers containing 800 ml of disinfectant solution. After 10 min, the machine was stopped and the feet were left hanging for another 60 min outside the disinfectant solution at room temperature. The dipping machine is supposed to imitate the situation of the sheep treated alive as described by Greber et al. [10]. In short, sheep are standing and moving their feet in the bath for 10 min. Foot movement is supposed to increase the contact with the disinfectant. After footbathing sheep are contained for 60 min on a clean and dry concrete floor.

A second interdigital swab (postvalue) was afterwards collected from the same area in the same way as described above. Prevalue and postvalue tubes were centrifuged for 5 min at 15'000xg and the supernatants were discarded. The remaining pellets were resuspended in 50 μl of 0.85% NaCl. The PMA-qPCR was performed as described before. Each swab sample was analyzed in duplicate, and the external standard was used for quantification.

For each foot, the reduction of live *D*. *nodosus* was calculated as follows:

$$\log_{10}R=\log_{10}\left(N\;prevalue\right)-\log_{10}\left(N\;postvalue\right)$$

With the exception of Desintec, all other substances from the *in vitro* experiment were not approved as biocide for use in footbaths with sheep. Therefore, only 3%, 6% and 9% Desintec was tested *ex vivo* and compared to 4% formaldehyde and 10% zinc sulfate. A 0.85% NaCl solution was chosen as negative control (Table 2).

**Table 2:** Disinfectants and concentrations tested *ex vivo* for their effect on reduction of live *D*. *nodosus*.

Disinfectant	Concentration	feet (n)
Formaldehyde	4%	13
Zinc sulfate	10%	12
DESINTEC® Hoof Care Special D	3%	12
	6%	14
	9%	14
NaCl	0.85%	12

The software NCSS12 (NCSS Statistical Software) was used for the statistical analysis of the logarithmized reduction values. Assumption of normal distribution was checked using histograms and Shapiro-Wilk test. The values were not normally distributed and equal variance was rejected. Therefore, Kruskal-Wallis Test with Dunn's Test for multiple comparisons was used for determination of significant differences among disinfectants. The significance level was calculated at z-value > 1.9600. Post hoc power testing was performed using Two-Sample T-Tests allowing unequal variance.

## Results

### *In vitro* disinfectant testing

Results for each disinfectant with the corresponding reduction of virulent *D*. *nodosus* are given in Table 3 for triplicate testing and S1 Table for single testing. The disinfectants formaldehyde (4%; 7.2/6.7) and copper sulfate (5%: 5.7/6.1; 10%: 7.2/6.7) achieved a ≥ 5 log reduction without (x/) and with (/x) organic soiling whereas 10% zinc sulfate (4.9/4.7) failed to meet the envisaged log reduction. The 20% zinc sulfate solution could not be evaluated, because centrifugation in the solution failed due to its high density.

**Table 3:** Effectiveness of the tested disinfectants on growth reduction of *D*. *nodosus*.

Trade name / Active ingredient	Concentrations tested	Log_10_ reduction without soiling^a^	Log_10_ reduction with soiling^a^
Formaldehyde	4%	7.2	6.7
Copper sulfate	5%	5.7	6.1
	10%	7.2	6.7
Zinc sulfate	10%	4.9	4.7
DESINTEC® Hoof Care Special D	1:10	6.8	6.8
	1:100	6.8	6.4
	1:1000	5.8	0.3
	1:10000	0.4	0.2
Acetic acid	5%	7.2	7.2
Glycolic acid	5%	7.7	6
Glutaraldehyde	5%	6.2	6.6

^a^ mean values of three replicates.

The individually tested active ingredients of Desintec at 5% (acetic acid; glycolic acid; glutaraldehyde) showed log reductions ≥ 5 (7.2/7.2; 7.7/6.0; 6.2/6.6) without and with organic soiling, respectively.

Desintec itself showed log reductions ≥ 5 in 1:10 and 1:100 dilutions without and with soiling (6.8/6.8 and 6.8/6.4, respectively). In a 1:1000 dilution, the product evoked a 5.8 log reduction without soiling and 0.3 log reduction with soiling whereas in 1:10 000 dilution both test conditions failed to meet the envisaged ≥ 5 log reduction (0.4/0.2).

In single experiments (S1 Table) also lactic acid, propionic acid, hydrogen peroxide, sodium hypochlorite, octenidine dihydrochloride, chlorocresol and Ampholyt 20 achieved the required ≥ 5 log reduction in the number of *D*. *nodosus* even under soiling conditions. While calcium magnesium tetrahydroxide, calcium hydroxide and calcium oxide showed the requested reduction without soiling but not with soiling, sodium benzoate, tartaric acid, calcium magnesium oxide and electrochemically activated water failed to meet the minimum ≥ 5 log reduction without and with soiling.

### *Ex vivo* experiments

#### Linear range of PMA-qPCR

**Fig 2:**
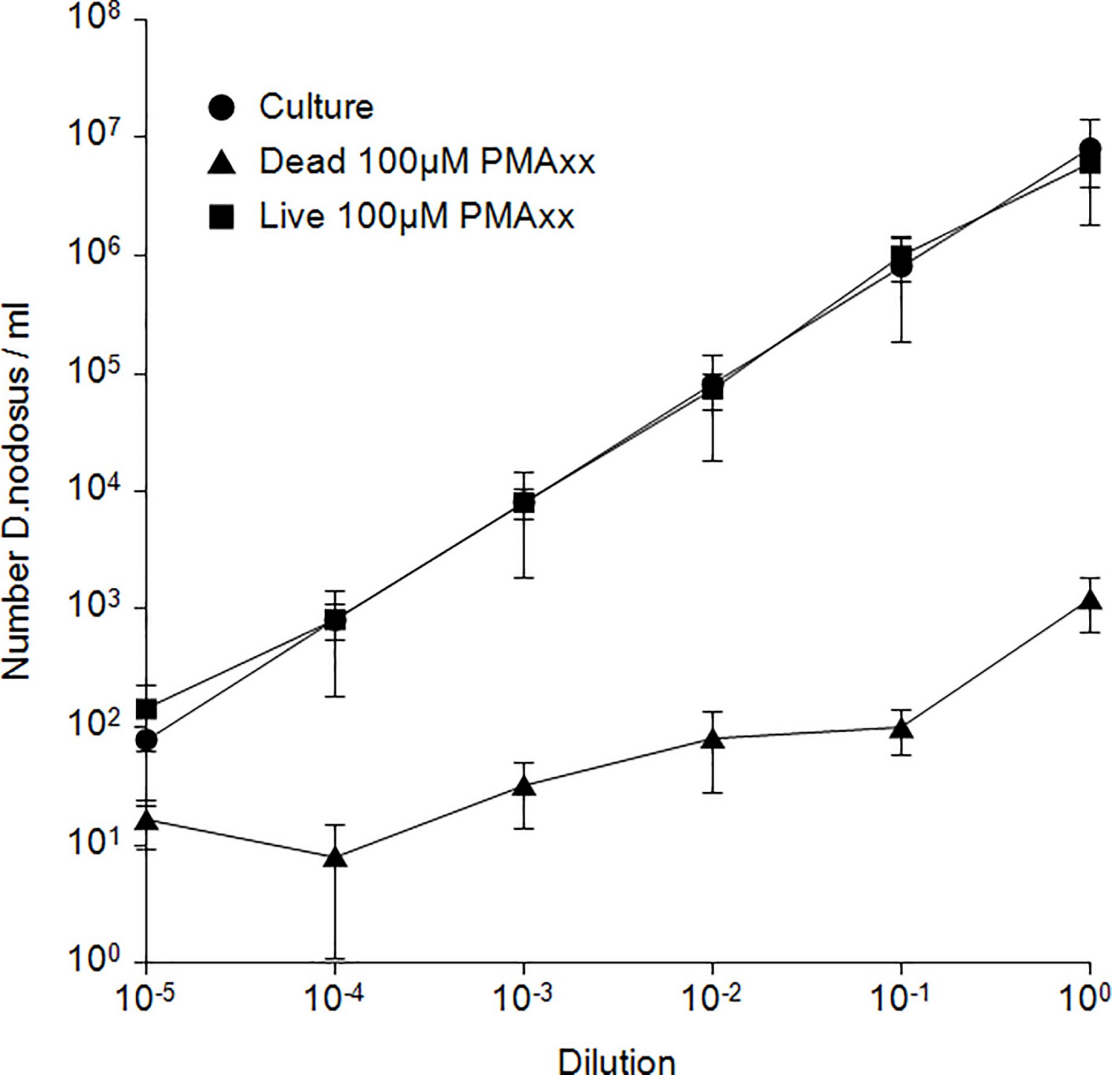
Number of viable and heat killed *D*. *nodosus* determined by culture and PMA-qPCR. Six mock samples of tenfold serial dilutions were used to demonstrate the linear range of established PMA-qPCR. Three independent runs were performed and average values calculated. Error bars represent standard deviation.

Enumeration of viable *D*. *nodosus* by culture and PMA-qPCR revealed good correlation (Fig 2). Samples containing 10^7^ to 10^3^ of *D*. *nodosus* showed a decrease of DNA in the dead cell sample by PMAxx^™^ treatment between 4 log and 2 log. At 10^2^ CFU/ml, the decrease dropped to 1 log unit. Therefore, part of the DNA corresponding to approximately 10^1^ to 10^3^ CFU/ml remained intact.

#### *Ex vivo* disinfectant testing

**Fig 3:**
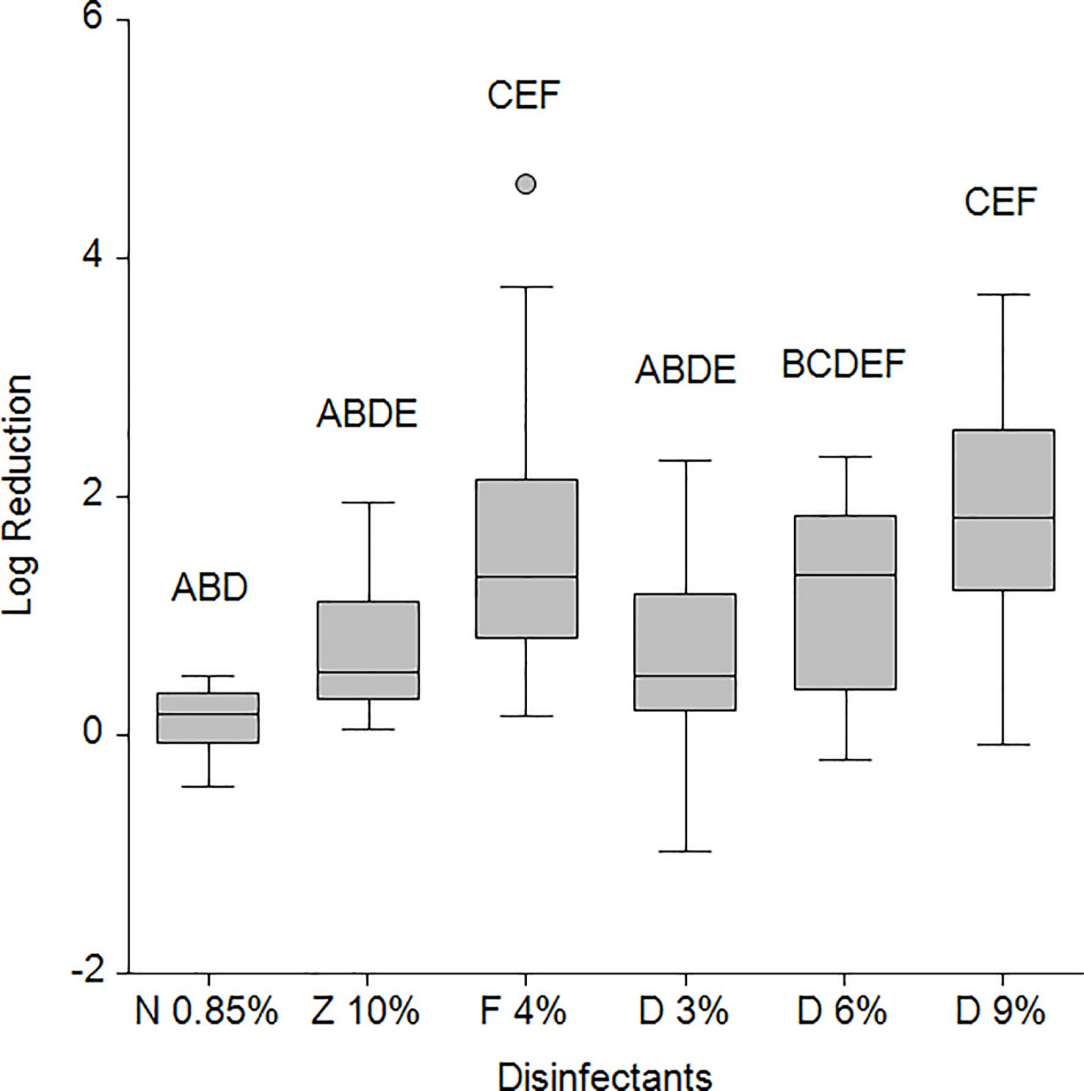
Efficiency of disinfectants in *ex vivo* experiments. Log-fold reduction of tested disinfectants against virulent *D*. *nodosus* determined by PMA-qPCR in the *ex vivo* experiments. Different superscript letters indicate significant differences in the mean at z-value >1.9600. N 0.85% (NaCl 0.85%), Z 10% (10% Zinc sulfate), F 4% (4% Formaldehyde), D 3% (3% Desintec), D 6% (6% Desintec), D 9% (9% Desintec).

Efficiency of disinfectants is shown in Fig 3. Medians of 10% zinc sulfate versus 0.85% NaCl were not different (z = 1.8705), whereas 4% formaldehyde evoked a significant reduction (z = 3.8794). For this reason, the more effective disinfectant formaldehyde was chosen for comparison with Desintec. Both 6% and 9% Desintec showed no significant differences compared to 4% formaldehyde (z = 0.8710 and 0.2646). Post hoc power testing between 4% formaldehyde and 6% Desintec with group sample sizes of 13 and 14 achieved 80.078% power to reject the null hypothesis of equal means when the population mean difference (delta) is 1 with standard deviations of 1.2 for group 1 and 0.8 for group 2, and with a significance level (alpha) of 0.050. A 4% formaldehyde solution is significantly more effective than 10% zinc sulfate (z = 1.9719). Unequal variances of disinfectants and a dose-responsive reduction of Desintec are visible in the box plot.

## Discussion

The aim of this study was to identify and test alternative disinfectants for future use as footbath solutions in a footrot control program. Based on the requirements for an effective, non-carcinogenic, environmentally acceptable, inexpensive and licensable concentrate, around 22 substances or products were selected by expert opinion. It became clear in discussions with representatives of veterinary drug companies that only an already registered product for footbaths would be considered for marketing since a new registration of a substance for the limited market would be too expensive. The product DESINTEC® Hoof Care Special D (Desintec) had already been registered as a biocide in Germany under the name PediSept G20 and became therefore the focus of the study. Thus, Desintec and its main components glutaraldehyde, acetic acid and glycolic acid, as well as the "gold standards" formaldehyde, copper sulfate, zinc sulfate and other chemical substances were tested.

The *in vitro* study confirmed the effectiveness of formaldehyde, copper sulfate and zinc sulfate as disinfectants in footbaths. These have been used for a long time and are an effective treatment option for footrot in sheep [10, 16]. However, the 10% zinc sulfate solution was slightly below the requested ≥5 log reduction and the 20% solution could unfortunately not effectively be tested due to its high density, which prevented *D*. *nodosus* and erythrocytes from sedimentation by centrifugation.

Desintec fulfilled the targeted reduction of ≥5 log at 1:10 and 1:100 dilution in accordance with the manufacturer recommendation to use it at a concentration of 3–5%. The product was even effective in 1:1000 dilution without organic soiling, however, organic soiling substantially reduced its effect. When testing the components of Desintec (glutaraldehyde, acetic acid, glycolic acid) individually at 5% concentration, each of them was able to reduce the number of *D*. *nodosus* at the requested scale even under soiling conditions.

Glutaraldehyde has a broad spectrum of activity and a rapid microbial killing rate. It is supposed to destroy all forms of microbial life, including bacterial and fungal spores, tubercle bacilli and viruses [17]. Glutaraldehyde is part of many disinfectant solutions for livestock, listed by the committee for disinfection of the German Veterinary Medical Society [18]. Organic acids are known to be used as food preservatives due to their antimicrobial potential [19]. Like that, acetic acid reduced the microbial load of foodborne pathogens on several fresh fruits and vegetables [20]. In medicine, acetic acid has been used for wound disinfection [21]. Even though microorganisms vary in their susceptibility, acetic acid solution proofed to be bactericidal for *D*. *nodosus* in the current study as well. Glycolic acid is well known for its keratolytic properties. The small molecular weight allows for easy penetration of the skin, targeting the corneosomes and resulting in desquamation of the stratum corneum [22]. *D*. *nodosus* can be found in a depth of 2200 μm in footrot affected tissue [23]. The keratolytic effect of glycolic acid may contribute to a deeper penetration of disinfecting ingredients of biocides into skin and hoof thus improving their effectiveness [24]. Apart from the keratolytic effect, glycolic acid shows antimicrobial properties like other carboxylic acids although it is less commonly used for this purpose. It proofed to be an effective postmilking teat disinfectant [25], and the strong reduction of *D*. *nodosus* in our *in vitro* experiments is another example of its antimicrobial activity.

Lactic acid, propionic acid, hydrogen peroxide, sodium hypochlorite, octenidine dihydrochloride, chlorocresol and Ampholyte 20 were effective in reducing the number of viable *D*. *nodosus in vitro* even under soiling conditions. On the other hand, calcium magnesium tetrahydroxide, calcium hydroxide and calcium oxide showed the requested reduction without soiling but not with soiling, while sodium benzoate, tartaric acid, calcium magnesium oxide and electrochemically activated water failed as disinfectant without and with soiling. Since all these components lack the chance of becoming registered biocides, they were only tested once and not considered in the *ex vivo* assays.

Based on the promising use of Desintec this product was further investigated in *ex vivo* tests using sheep feet in a dipping machine (Fig 1). In contrast to the *in vitro* assay, culturing of *D*. *nodosus* from a swab sample of heavily contaminated feet is not a sensitive method and requires subculturing, which makes a quantification of viable *D*. *nodosus* as needed for assessing effectiveness of disinfectants in the *ex vivo* part of our study impossible. Therefore, an alternative approach able of detecting viable cells by PCR as e.g. presented by Nogva et al. [26] was established. By this approach, the distinction between viable and non-viable cells is possible, based on membrane integrity. For that purpose, the samples containing *D*. *nodosus* were treated with the improved nucleic acid intercalating propidium monoazid (PMA) dye PMAxx^™^ that selectively enters cells with compromised cell membranes, whereas the intact cell membrane presents a natural barrier for this molecule. After exposure to strong light, it covalently binds to the DNA, preventing DNA from being amplified by PCR, thereby enabling differentiation of viable from non-viable cells. At the same time when the cross-linking with DNA occurs, any unbound excess PMAxx^™^ reacts with water. The resulting molecule is no longer reactive, preventing reaction of PMAxx^™^ with DNA extracted from intact cells [27].

The PMA-qPCR proved to be a valid method for comparison of antimicrobial efficiency in the *ex vivo* experiments. Thereby, 10% zinc sulfate did not show a significant difference to the NaCl control. Therefore, 4% formaldehyde that showed a significant reduction of live *D*. *nodosus* was chose to compare to Desintec. The Desintec solution diluted down to 6% was still effective not showing any significant difference to the "gold-standard" of 4% formaldehyde. Variability of reduction within disinfectant groups was observed and is explainable by various factors influencing the *ex vivo* experiment. *D*. *nodosus* loads vary naturally among feet due to individual differences and the different clinical status of footrot affected feet, leading to different prevalues [28]. Furthermore, the total number of microorganisms can also affect the activity of a disinfectant. Higher inoculum levels can attenuate the efficacy of disinfection by adding to the level of soiling and by providing protection to other organisms at the site [29]. In addition, disinfection inactivation follows first-order kinetics. Starting from a high prevalue, the absolute reduction is high whereas the relative reduction is small. On the other hand, starting from a small prevalue, the effect is reversed with small absolute and high relative reduction. Reduction values obtained from smaller prevalues possibly lead to higher relative reductions, widening the variability. Negative reduction can be explained with the low precision of the swab sample. Another reason for the variability could be the extent of soiling. Soiling can affect both the disinfection process and PMA-qPCR. Even though claws and interdigital space were cleaned and open articulations were covered, disinfectant solutions were soiled with manure and blood to varying degrees after the footbath. Soiling can affect microbial activity by direct interference with the disinfectant, by interaction and protection of the target organism and formation of microbial aggregates [30]. Moreover, complex matrices found in environmental samples can negatively influence the PMAxx^™^ treatment by chemical adsorption of the dye and interference with photoactivation [27].

Glutaraldehyde contained in Desintec is not listed by the International Agency for Research on Cancer (IARC) and there is no evidence for carcinogenic activity, genetic or reproductive toxicity. However, glutaraldehyde is irritating and corrosive to the skin, eyes and respiratory tract and is a known cause of allergic contact dermatitis and occupational asthma [31]. It is toxic to aquatic life and should not be discharged into water bodies [32]. On the other hand, a smaller amount of glutaraldehyde in combination with acetic and glycolic acid is needed to achieve the same antimicrobial effect as 4% formaldehyde. In a 6% Desintec solution (recommended final concentration) there is 0.36% glutaraldehyde compared to 4% formaldehyde currently used in footbaths. Furthermore, glutaraldehyde is rapidly degradable in air, water, and soil, does not bioaccumulate and is less toxic than formaldehyde [33–35]. Desintec applied as 6% solution in footbaths is therefore an environmentally acceptable biocide that after use is recommended to be disposed on the manure pile or in the slurry basin.

## Conclusion

The study showed that Desintec is an effective alternative to formaldehyde (4%), zinc sulfate (10%) and copper sulfate (5%) for the use in sheep footbaths to eliminate virulent *D*. *nodosus*. The product is not only effective but also non-carcinogenic, is biodegradable and available as concentrate, making it an improvement over the currently used disinfectants. The results of this study represent a step forward on the way to a footrot control program that will mainly be based upon herd-level footbathing.

## Supporting information

S1 Table Effectiveness of disinfectants tested in single experiment on growth reduction of *D*. *nodosus*. (DOCX)
