# Supplementary material for: In vitro and ex vivo testing of alternative disinfectants to currently used more harmful substances in footbaths against Dichelobacter nodosus
Source: PLoS One. 2020 Feb 13;15(2):e0229066. doi: 10.1371/journal.pone.0229066 (PMC7018501; doi:10.1371/journal.pone.0229066)
Supplement: S1 Table — (DOCX) [file pone.0229066.s001.docx]

S1 Table. Effectiveness of disinfectants tested in single experiment on growth reduction of *D. nodosus*

**Trade name / Active ingredient Concentrations tested Log_10_ reduction without soiling Log_10_ reduction with soiling**

L-Lactic acid 5% 7.2 7.2

Sodium benzoate 5% 0.2 n.d.

Propionic acid 5% 7.2 7.2

Tartaric acid 5% 4.9 n.d.

Calcium magnesium oxide max. solubility (0.11%) 1.5 n.d.

Calcium magnesium tetrahydroxide max. solubility (0.99%) 7.07 1.2

Calcium hydroxide max. solubility (0.136%) 7.07 1.4

Calcium oxide max. solubility (0.076%) 5.69 1

**Desical** ® **plus** max. solubility (0.112%) 0.4 n.d.

Hydrogen peroxide 5% 7.2 7.2

Sodium hypochlorite 5% 6.2 6.6

**Toucan Eco ®** undiluted 0.3 n.d.

Octenidine dihydrochloride 5% 6.4 6.3

Chlorocresol max. solubility (0.4%) 6.4 6.3

**Tego ® 2000 VT25 (**Ampholyt 20) 5% 6.2 7.2

n.d., not determined
